# Measuring diversity in medical reports based on categorized attributes and international classification systems

**DOI:** 10.1186/1472-6947-12-31

**Published:** 2012-04-12

**Authors:** Petra Přečková, Jana Zvárová, Karel Zvára

**Affiliations:** 1 Centre of Biomedical Informatics and EuroMISE Center, Institute of Computer Science AS CR, Pod Vodárenskou věží 2, Prague 8 182 07, the Czech Republic

## Abstract

**Background:**

Narrative medical reports do not use standardized terminology and often bring insufficient information for statistical processing and medical decision making. Objectives of the paper are to propose a method for measuring diversity in medical reports written in any language, to compare diversities in narrative and structured medical reports and to map attributes and terms to selected classification systems.

**Methods:**

A new method based on a general concept of f-diversity is proposed for measuring diversity of medical reports in any language. The method is based on categorized attributes recorded in narrative or structured medical reports and on international classification systems. Values of categories are expressed by terms. Using SNOMED CT and ICD 10 we are mapping attributes and terms to predefined codes. We use f-diversities of Gini-Simpson and Number of Categories types to compare diversities of narrative and structured medical reports. The comparison is based on attributes selected from the Minimal Data Model for Cardiology (MDMC).

**Results:**

We compared diversities of 110 Czech narrative medical reports and 1119 Czech structured medical reports. Selected categorized attributes of MDMC had mostly different numbers of categories and used different terms in narrative and structured reports. We found more than 60% of MDMC attributes in SNOMED CT. We showed that attributes in narrative medical reports had greater diversity than the same attributes in structured medical reports. Further, we replaced each value of category (term) used for attributes in narrative medical reports by the closest term and the category used in MDMC for structured medical reports. We found that relative Gini-Simpson diversities in structured medical reports were significantly smaller than those in narrative medical reports except the "Allergy" attribute.

**Conclusions:**

Terminology in narrative medical reports is not standardized. Therefore it is nearly impossible to map values of attributes (terms) to codes of known classification systems. A high diversity in narrative medical reports terminology leads to more difficult computer processing than in structured medical reports and some information may be lost during this process. Setting a standardized terminology would help healthcare providers to have complete and easily accessible information about patients that would result in better healthcare.

## Background

We can consider two approaches how to store information about health state of patients in a medical report. The first one is based on storing information in the form of a free text and thus creating narrative medical reports. The second approach is to store information in a structured form (e.g. using structured electronic health record) and thus create structured medical reports.

In current medicine we can meet with many synonyms for one concept, e.g. single disease. That was the reason why coding systems providing codes for any medical findings have arisen. Coding systems limit the variability of expression. Only the approved terms and their phrases can be used according to strictly given rules. Formal codes are usually used instead of the approved terms. In many cases it is useful if coding systems show also not approved terms, which are often used as synonyms for approved terms.

Among the most widespread international classification systems can be ranked: International Classification of Diseases and Related Health Problems (ICD), Systematized Nomenclature of Medicine Clinical Terms (SNOMED CT), Medical Subject Headings (MeSH), Logical Observations Identifiers Named and Codes (LOINC) and others, more than 100 classifications systems.

**ICD **is one of the oldest medical classification systems. The foundation was laid in 1855. The World Health Organization took it over in 1948. At that time it was its 6th revision. Since 1994 the 10th revision of ICD is in use and it contains 22 chapters [1-3]. ICD has become an international standard for a classification of diseases and for many epidemiological and management needs in healthcare. These include a general situation of health in different population groups and monitoring of the incidence and prevalence of various diseases and other health problems in relation to other variables. It is used to classify diseases and other health problems that are recorded in many types of health records, including death certificates and hospital records. ICD is available in six official languages of WHO and in other 36 languages, including Czech.

**SNOMED CT **[4-12] is a comprehensive clinical terminology that provides clinical content and expressivity for clinical documentation and reporting. It can be used to code, retrieve, and analyze clinical data. SNOMED CT resulted from the merger of SNOMED Reference Terminology developed by the College of American Pathologists and Clinical Terms Version 3 developed by the National Health Service of the United Kingdom. The terminology is comprised of concepts, terms and relationships with the objective of precisely representing clinical information across the scope of health care. SNOMED CT provides a standardized clinical terminology that is essential for effective collection of clinical data, its retrieval, aggregation and re-use, as well as interoperability. SNOMED CT is considered to be the most comprehensive, multilingual clinical healthcare terminology in the world. Since April 2007 is owned, maintained, and distributed by the International Health Terminology Standards Development Organisation, a not-for-profit association in Denmark. Nowadays we can meet with American, British, Spanish, and German versions of SNOMED CT.

**MeSH **[13,14] is a vocabulary controlled by the National Library of Medicine (NLM). It is composed of terms, which denominate keywords hierarchically and this hierarchy helps with searching on various levels of specificity. Keywords are arranged not only alphabetically but also hierarchically. NLM uses MeSH for indexing of papers from world best biomedical journals for the MEDLINE/PubMED database. MeSH is used also for a database cataloguing books, documents, and audiovisual materials. Each bibliographical reference is connected with a class of terms in the MeSH classification system. Searching inquiries use also the MeSH vocabulary to find papers with required topics. There exists also the Czech translation of MeSH.

**LOINC **[15,16] is a clinical terminology, which is important for laboratory tests and laboratory results. In the year 1999 the HL7 organization accepted LOINC as a preferred coding system for names of laboratory tests and clinical observations.

The increasing number of classification systems and nomenclatures requires designing of various conversion tools for transfer among main classification systems and for recording of relations among terms in these systems. Extensive ontologies and semantic networks are modeled for information transfer among various databases. Metathesauri are designed to monitor and connect information from various heterogeneous sources. The most extensive is the Unified Medical Language System (UMLS) [17-19], which we used in our study.

As it was stated in [20-22], use of new metaterms as terminological tools was able to facilitate information retrieval from a medical record system.

The Czech language belongs to the western group of Slavic languages and it belongs to the free constituent order languages [23]. The style of writing narrative medical reports in the Czech Republic, as in any other country, is not standardized and they are written mostly in the form of a free text [24]. Similarly as in other languages, Czech narrative medical reports do not use standardized terminology and they often bring insufficient information for statistical processing and medical decision making. For one term we can often meet with more than ten synonyms. Synonyms in narrative reports lead to inaccuracy and to misunderstanding. This problem has intensified with an introduction of a computer technology to healthcare. Using computers means higher uniqueness of data feeding, of term definitions, their precise denomination, etc., thereby the significant drawback becomes more noticeable. Generally, in the scientific terminology it is more advantageous to use only one expression for one term. Computers are able to learn synonyms but it enlarges dictionary databases and the number of necessary operations grows. Moreover, standardized terminology is a basic assumption for semantic interoperability.

## Methods

One of our objectives was to propose a new application for measuring diversity of medical reports written in any language. The method is based on categorized attributes recorded in medical reports and on international classification systems. The general concept of diversity is derived from f-diversity and its modifications (relative f-diversity, self f-diversity and marginal f-diversity). Here we use f-diversities of Gini-Simpson and Number of Categories types. The method can be applied to compare diversities of two samples of medical reports. We compared diversities of the samples of Czech narrative medical reports and Czech structured medical reports. Both samples were collected in two outpatient departments of preventive cardiology of the Municipal Hospital in Čáslav. The first outpatient department was located in Prague and the second one in Čáslav. The Municipal Hospital in Čáslav approved using these medical reports for our research. We used categorized attributes selected from the Minimal Data Model for Cardiology (MDMC). Medical reports were recorded by four physicians. We analyzed 1119 structured medical reports collected in Prague and 110 narrative medical reports collected in Čáslav. We included narrative medical reports from Čáslav collected by the same physicians that collected also data for structured medical reports in Prague.

### Minimal data model for cardiology

Nowadays, there is a big boom in the development of electronic health records (EHRs). There is a general agreement that EHR has the potential to improve quality of medical care [25]. The most important seems to be the requirement on EHRs for exchanging and management of structured health information. For our study the field of cardiology has been chosen because since 1994 the EuroMISE Centre [26] has been running two outpatient departments of preventive cardiology under the auspices of the Municipal hospital of Čáslav and therefore we have access mainly to the cardiological data and medical records focused on cardiology. In 2002 the Minimal Data Model for Cardiology (MDMC) was developed within this research center [27,28]. MDMC is a set of approximately 150 attributes, their categorization, mutual relations, integrity restrictions, units, etc. Prominent professionals in the field of Czech cardiology agreed on these attributes as on the basic data necessary for an examination of a patient in cardiology.

MDMC consists of eight groups of attributes. The first one is the *administrative part*. Then, there is a *family history part *with information on parents and siblings. The next part is the *social history and addiction *focusing on the marital status, physical activities, mental stress, levels of smoking and alcohol consumption rates. One part of MDMC is devoted to *allergies*, mainly to drug allergies. The *personal history part *detects the presence of diabetes mellitus, there is observed whether a patient suffered from a stroke, whether he/she is treated with an ischemic disease of periphery arteries, there are attributes related to aortic aneurysm, other relevant diseases and menopause in women. In the part called *Current difficulties of a possible cardiological origin *physicians focus on shortness of breath, chest pain, palpitations, swellings, syncope, cough, hemoptysis, and claudication. Another part determines what kind of a *treatment *a patient undergoes, what type of a diet is prescribed and which medications he/she uses. In the part of the *physical examination*, patient's weight, height, body temperature, BMI, WHR, blood pressure, pulse and breathing rates, and pathological findings are determined. *Laboratory testing *is focused on blood glucose, uric acid, total cholesterol, HDL-cholesterol, LDL-cholesterol, and triaglycerols. The last part is focused on attributes related to *ECG*. The beat frequency, the average PQ and QRS intervals and results of ECG are fully described there.

### Traditional measures of diversity

Traditional measures of diversity are based on categorized attributes. For a given attribute we determine categories *A_1_,..., A_k-1_*. Then, we summarize the rest findings in the "others" category and we denote this category as *A_k_*. The most known two measures of diversity are the following.

The **Gini-Simpson index ***H_GS _*(**p**) is calculated from the probability distribution **p **= (*p*_1_,..., *p_k_*) of *k *categories of a given attribute as

(1) $$H_{GS}\left(p\right)=1-\overset{k}{\underset{i=1}{\sum }}p_{i}^{2}.$$

The Gini-Simpson index has its values in the interval [0, (*k *- 1)/*k*], where the lower boundary 0 is reached if and only if there is only one category of the studied attribute and the upper boundary (*k -*1)/*k *for **p **= **u **= (1/*k*, 1/*k*,..., 1/*k*) for uniform probability distribution. Originally it was suggested as a measure of inequality in income by Gini [29] and later discussed by Simpson [30] as a measure of ecological diversity.

The second one, the **Shannon information index ***H_S _*(**p**), is calculated from *p_1_,..., p_k _*probabilities of *k *categories of a given attribute as

(2) $$H_{s}\left(p\right)=-\overset{k}{\underset{i=1}{\sum }}p_{i}\text{log}p_{i}$$

The Shannon information index has its values in the interval [0; log *k*], where the lower boundary 0 is reached if and only if there is only one category of the attribute and the upper boundary log *k *for uniform probability distribution **p **= **u **= (1/k,..., 1/k).

It is hard to give a universal preference to one of these two measures. Some researchers are more familiar with the Shannon entropy and it is easier for them to interpret particular numerical values of *H_S _*(**p**) than those of *H_GS _*(**p**). On the other hand, the Gini-Simpson index is a very well-known traditional measure of diversity.

### f-diversity and relative f-diversity

Shannon information *I_S _*(*X; Y*) is defined in information theory as a measure of an association between two attributes *X *and *Y*.

(3) $$I_{s}\left(X;Y\right)=\underset{x,z}{\sum }p\left(x,y\right)\text{log}\frac{p\left(x,y\right)}{p\left(x\right).p\left(y\right)},$$

where *p*(*x; y*) are the joint probabilities and *p*(*x*); *p*(*y*) marginal probabilities of categories of *X *and *Y *attributes.

Shannon information *I_S _*(*X*; *Y*) is nonnegative and equal to zero if and only if the attributes are independent. Maximal information is the Shannon entropy obtained if *Y *= *X*. In case that the attribute *X *has categories *A_1_, A_2_,..., A_k _*occurring with probabilities *p_1_, p_2_,..., p_k _*respectively, then the **Shannon entropy **of the attribute *X *is the same as the Shannon information index

$$H_{s}\left(p\right)=-\overset{k}{\underset{i=1}{\sum }}p_{i}\text{log}p_{i}.$$

This measure of diversity will be further called **Shannon diversity**.

Shannon information can be generalized to the f-information

(4) $$I_{f}\left(X,Y\right)=\underset{x,y}{\sum }\left(\frac{p\left(x,y\right)}{p\left(x\right).p\left(y\right)}\right)p\left(x\right).p\left(y\right)$$

where *f(t) *is a convex function on the interval [0;∞), strictly convex at *t = *1 with *f*(1) = 0. For more details about f-information derived from the concept of f-divergence see Vajda [31]. In case of *f(t) *= *t *log *t*, f-information *I_f _*(*X; Y*) reduces to Shannon information *I_S_*(*X; Y*) that is widely used in pattern recognition and decision support, see e.g. [32-35]. For the first time f-information was systematically studied by Zvárová [36] who proved the representation of maximal f-information and called it f-entropy. In case that *X *is an attribute with categories *A_1; _A_2_;..., A_k _*and probability distribution ***p ***= (*p_1_, p_2_,..., p_k_*), then **f-entropy **of the attribute *X *is

(5) $$H_{f}\left(p\right)=\overset{k}{\underset{i=1}{\sum }}p_{i}^{2}f\left(1/p_{i}\right)+f\left(0\right)\overset{k}{\underset{i=1}{\sum }}p_{i}\left(1-p_{i}\right).$$

f-entropy H_f _(**p**) can be interpreted as an average unpredictability of the individual categories *A_i _*of the attribute *X *[37]. In this sense f-entropy H_f _(***p***) is a measure of diversity depending on the distribution **p**. H_f _(***p***) will be called **f-diversity **if it moreover satisfies the following conditions:

• H_f _(**p**) is non-negative,

• H_f _(**p**) reaches its minimal value in case that there is one category with probability 1,

• H_f _(**p**) reaches its maximal value in case that **p **= **u **is the uniform distribution,

• H_f _(**p**) is a symmetric function of ***p***,

• H_f _(**p**) is a concave function on the system of all probability distributions **p**.

We can see that H_f _(**p**) is a sum of two expressions where the second one is nothing but the well-known Gini-Simpson index H_GS _(**p**) multiplied by the constant *f*(0). Further we will call Gini-Simpson index the **Gini-Simpson diversity**. In the paper [36] it was proved that f-diversities can be found among f-entropies satisfying the condition *g*(*t*) = (*f*(*t*) - *f*(0))/*t *is a concave function. Then f-entropy H_f_(**p**) of the attribute *X *will reach its maximal value for uniform distribution of categories ***p ***= **u**. We can see that Gini-Simpson diversity H_GS _(**p**) is f-diversity with *f*(*t*) = *t *- 1 for *t *> 1, otherwise *f*(*t*) = 0. Similarly, Shannon diversity is f-diversity with *f*(*t*) = *t *log *t*.

**Relative f-diversity **RH_f _(**p**) was defined in [37] as f-diversity H_f _(**p**) divided by f-diversity of the uniform distribution H_f _(**u**) as

$$\text{R}{\text{H}}_{\text{f}}\left(p\right)={\text{H}}_{\text{f}}\left(p\right)/{\text{H}}_{\text{f}}\left(u\right).$$

### Measures of rarity, self and marginal f-diversity

In case that *X *is an attribute with categories *A_1_*,..., *A_k _*and a probability distribution **p **= (*p*_1_,..., *p_k_*), then according to Patil and Tailie [38] the rarity of the category *A_i _*depends only on the numerical value of *p_i_*. Denoting the rarity of the category *A_i _*by *R(p_i_) *the **diversity index **associated with the measure of rarity *R *is its average rarity calculated as

(6) $$\overset{k}{\underset{i=1}{\sum }}p_{i}R_{i}\left(p\right).$$

Three widely used diversity indexes are:

Number of categories (Number of categories diversity)

(7) $$H_{NA}=k-1\;\text{with}\;R_{NA,i}\left(p\right)=\left(1-p_{i}\right)/p_{i}$$

Gini-Simpson index (Gini-Simpson diversity)

(8) $$H_{GS}\left(p\right)=\overset{k}{\underset{i=1}{\sum }}p_{i}\left(1-p_{i}\right)\text{with}\;R_{GS,i}\left(p\right)=1-p_{i}$$

and Shannon index (Shannon diversity)

(9) $$H_{S}\left(p\right)=\overset{k}{\underset{i=1}{\sum }}p_{i}\text{log}p_{i}\text{with}\;R_{S,i}\left(p\right)=-\text{log}p_{i}.$$

These three diversity indexes belong to the family of diversity indexes of order *β *[38] defined as

(10) $$R_{i}\left(p_{i}\right)=\left\{\begin{array}{c} \left(1-p_{i}^{\beta }\right)/\\ -\text{log}p_{i} \end{array}.\right)\begin{array}{c} if\beta \geq -1,\beta \neq 0\\ if\beta =0. \end{array}$$

We can see that for *β *= 0 we receive the Shannon diversity, for *β *= 1 the Gini-Simpson diversity and for *β *= -1 the Number of categories diversity. As it was shown above, all of these three diversity indexes belong to the family of f-diversities.

Let us introduce the concept of self f-diversity [39] that is a generalization of the rarity introduced by Patil and Tailie [38]. **Self f-diversity **of the *i*-th category is defined as

(11) $$R_{f,i}\left(p\right)=p_{i}f\left(1/p_{i}\right)+f\left(0\right)\left(1-pi\right)$$

Then it can be proved that f-diversity can be calculated from self f-diversities as

(12) $$\begin{array}{c} H_{f}\left(p\right)=\overset{k}{\underset{i=1}{\sum }}p_{i}\left(p_{i}f\left(1/p_{i}\right)+f\left(0\right)\left(1-p_{i}\right)\right)\\ =\overset{k}{\underset{i=1}{\sum }}p_{i}R_{f,i}\left(p\right). \end{array}$$

Therefore f-diversity ***H**_f _*(**p**) is the weighted average of self f-diversities *R_f,i_*(***p***).

For the often used Shannon diversity the **Shannon self diversity **is equal to

(13) $$R_{s,i}\left(p\right)=-\text{log}\left(p_{i}\right)$$

known in information theory also as **self information**. Similarly, for the Gini-Simpson diversity the **Gini-Simpson self diversity **is equal to

(14) $$R_{GS,i}\left(p\right)=1-p_{i}.$$

Another view on the impact of the *i*-th category is opened if we will not distinguish among other categories. In this case we formally work with two categories (dichotomy) with probabilities *p_i _*and 1- *p_i_*. Then **marginal f-diversity **of the *i*-th category is defined as

(15) $$H_{f,i}\left(p\right)=p_{i}^{2}f\left(1/p_{i}\right)+\left(1-p_{i}\right)f\left(1/\left(1-p_{i}\right)\right)+2f\left(0\right)p_{i}\left(1-p_{i}\right).$$

Next, we introduce relative self diversity and relative marginal diversity. We define the **relative self- diversity **of the *i*-th category as

(16) $$RR_{f,i}\left(p\right)=R_{f,i}\left(p\right)/H_{f}\left(p\right)$$

and the **relative marginal diversity **of the *i*-th category we define as

(17) $$RH_{f,i}\left(p\right)=H_{f,i}\left(p\right)/H_{f}\left(\tilde{u}\right),$$

where $\tilde{u}=\left(\frac{1}{2},\frac{1}{2}\right)$.

Comparing diversities on the sample of 110 Czech narrative medical reports and 1119 Czech structured medical reports we used diversities of the Gini- Simpson type. The reason is that there was shown in [40] that an ideal estimator of the Shannon type diversity does not exist.

## Results

MDMC has become a basis for selection of attributes and their categorization.

The analysis of suitability and utilizability of individual terminological thesauruses has been started by mapping clinical contents of the Minimal Data Model for Cardiology to various terminological classification systems.

First of all, we have tried to map the attributes and terms of MDMC to the SNOMED CT system. The first prerequisite for this mapping was the translation of the MDMC attributes and terms to the English language as there is not a Czech version of SNOMED CT [41].

As ICD-10 is one of a few international medical classifications translated to the Czech language, as the second step, we have tried to map the attributes and terms of MDMC to this ICD-10. Results of these mappings were published in [42].

We found the following types of MDMC attributes and terms from the point of view of possibilities of their mapping to SNOMED CT and ICD-10 classification systems:

• *Trouble-free terms and attributes *- i.e. terms and attributes, which can be mapped directly, so only one possibility of mapping exists; possibly there are only synonyms with exactly same meanings and therefore the same classification code (e.g. *patient first name, current smoker, motility, height of a patient*, etc.).

• *Partially problematic terms and attributes *- i.e. terms and attributes, which can be mapped in a way that there are several possibilities of mapping to different synonyms, which differ slightly in their meanings and usually in their classification codes (e.g. *ischemic cerebro-vascular stroke, angina pectoris, hypertension, congestive cardiac failure*, etc.).

• *Terms and attributes with a too small granularity - *i.e. terms and attributes describing certain characteristics on a too general level so that classification systems contain only terms of a narrower meaning (e.g. *e-mail *in MDMC versus *e-mail to work/e-mail to home/e-mail of a physician *and so on in classification systems).

• *Terms and attributes with a too big granularity *- i.e. terms and attributes describing certain characteristics on such a narrow level so that classification systems contain only a term of a more general meaning (e.g. *symmetrical pulse of carotids*, etc.).

• *Terms and attributes, which cannot be found in classification systems*, e.g. *dyslipidemy*, etc.

### Linguistics and lexical analysis of narrative medical reports

In the following part we present our findings on linguistic and lexical differences in Czech narrative medical reports.

#### Diacritic

The Czech language uses the diacritical writing system. As an example of diacritical letters let us mention e.g. letters "ě, č, ř, ž". However, it is faster for physicians to write without these diacritical marks and use letters "e, c, r, z". Such a text is for Czech native speakers understandable but it is difficult for computational processing.

#### Typing errors

Typing errors represent a bigger problem and they are very frequent in any language. The text is then very hardly usable for computational processing.

#### Spaces

A similar problem is spaces omitting between words, which results in merging of two words in one.

#### Figure 0

For computational processing it is difficult if a physician uses the figure 0 instead of the capital letter O.

#### Abbreviations

As physicians are often pressed of time they abbreviate words while writing medical reports. Unfortunately, there exists not a single rule how particular attributes should be abbreviated. Therefore the same words can be abbreviated diversely.

#### Rounding-off

Many discrepancies are connected with numerical values. One physician may round one attribute to integers, while another rounds the same attribute with the precision of one or two decimal numbers. Sometimes numerical values are presented as ranges, e.g. "70-80". Often only an approximate indication is entered, e.g. "diastolic pressure around 70". Some attributes are not expressed in numbers but in words, e.g. "blood pressure is within the normal range".

#### Arabic and Roman numerals

There is a divergence in usage of Arabic and Roman numerals. For example heart sounds may be found in both ways "heart sounds 2" and "heart sounds II".

#### Synonyms

The Czech language is very rich in synonyms and they are highly used also in medical reports.

#### Orthography

Some physicians use a newer version of Czech spelling, the others the older one.

#### Time data

Recording of time is not standardized as well. In medical reports we can run into the name of the month, e.g. "February 2006" but also the month order, e.g. "2/2006".

#### Drugs administering

There are various ways how to describe the time when a patient should administer a drug (e.g. 1-0-0 vs. 1 pill in the morning vs. 1 in the morning vs. 1× in the morning).

These are not problems only of writing medical reports but the same orthographic errors may be found e.g. in web pages [43].

### Mapping MDMC attributes to international classification systems

After translating the MDMC attributes from Czech to English, we have found that more than 60% of MDMC attributes could be mapped to SNOMED CT [42].

As the second step, we mapped the MDMC attributes to ICD-10. As the very title of the International Classification of Diseases shows, this classification can be used to encode particular diseases, syndromes, pathological conditions, injuries, difficulties and other reasons for the contact with healthcare services, i.e. the type of information that is being registered by a physician. Unfortunately, using this classification we cannot map many attributes of the MDMC, such as marital status, education, mental stress, physical stress, physical activity, smoking, alcohol drinking, physical examination (weight, height, body temperature, BMI, WHR, etc.) or laboratory tests (total cholesterol, HDL-cholesterol). ICD-10 can be used only for the parts of MDMC related to personal history and current difficulties of a possible cardiological origin. Therefore only 25% of MDMC has been mapped to ICD-10 [42].

Similar results were achieved when analyzing standardization possibilities of attributes of the Data Standard of Ministry of Health of the Czech Republic (DASTA) [44], in which the majority of healthcare information systems in the Czech Republic communicate. DASTA is based on the national classification system called the National code-list of laboratory items (NCLP) [44]. These standards are developed and administered by the developers of healthcare information systems that are specialized companies, universities or research institutions in the Czech Republic. The development of the standard is supported by the Czech Ministry of Health. DASTA is specialized mainly in transfer of requests and results of laboratory analyses. The current version of DASTA is XML based and provides also the functionality for sending statistical reports to the Institute of Health Information and Statistics of the Czech Republic [45] and limited functionality of free text clinical information exchange. Unfortunately, DASTA has almost no relation to international communication standards such as HL7 [46] or European standards like EN13606 [47].

### Diversities of selected attributes and their categories in narrative and structured medical reports

We analyzed 110 Czech narrative medical reports from the outpatient department in Čáslav and 1119 Czech structured medical reports from the outpatient department in Prague. In Table 1 we summarized results of the analysis of the same selected attributes collected in narrative and structured medical reports. Categorization of attributes was done according to MDMC in 1119 structured medical reports and categories were created as values of attributes recorded in free text of 110 narrative medical reports. As mentioned above, we found numbers of categories for selected attributes in narrative medical reports and we calculated the Number of categories diversity (Table 1).

**Table 1:** Number of categories

Attribute	No. of narrative reports with recorded attribute	No. of narrative reports with missing attribute	Number of categories diversity (narrative reports)	No. of structured reports with recorded attribute	No. of structured reports with missing attribute	Number of categories diversity (structured reports)
Smoking	71	39	29	1080	39	3

Allergy	90	20	12	1065	54	2

Ischemic heart disease	67	43	12	1045	74	2

Dyspnoea	79	31	28	934	72	2

Chest pain	38	72	16	1049	70	2

Palpitations	17	93	7	1054	65	1

Swelling	95	15	25	1050	69	1

Diabetes mellitus	69	41	14	1073	46	1

Number of categories for selected attributes found in 110 narrative reports and number of MDMC categories in 1119 structured medical reports

Each narrative medical report was read and analysed individually, one by one, and all various ways of describing selected MDMC attributes were highlighted and recorded. As there were much more ways describing these attributes in narrative reports than in the structured reports categorized according to MDMC, we can see that the Number of categories diversity is much higher in narrative medical reports than in structured medical reports.

Further, we transformed categories of attributes created from narrative reports by assigning each category of narrative reports the closest MDMC category. We estimated probabilities of MDMC categories for all attributes from narrative and structured medical reports without missing observations and calculated estimates of Gini-Simpson diversities (Table 2), Gini-Simpson self diversities and relative marginal diversities (Table 3). As we can see from (14), the Gini-Simpson self diversity of a category is expressed as probability of its complementary category. Therefore with decreasing probability of a category the Gini-Simpson diversity is increasing. The sum of all Gini-Simpson self diversities for a given attribute is equal to the number of its categories minus one. The Gini-Simpson relative marginal diversity of a category is increasing when probability of a category is approaching to $\frac{1}{2}$. In case that a selected attribute has only two categories, then Gini-Simpson relative marginal diversities of these two categories have the same value.

**Table 2:** Gini-Simpson relative diversities

Attribute	No. of categories	Number of narrative reports	Gini-Simpson relative diversity	Number of structured reports	Gini-Simpson relative diversity	p value
Smoking	4	71	0.8162	1080	0.6579	0.00073

Allergy	3	90	0.6300	1065	0.6977	0.30932

Ischemic heart disease	3	67	0.4117	1045	0.1455	0.00359

Dyspnoea	2	79	0.9152	1047	0.3851	< 0.00001

Chest pain	2	38	0.7756	1049	0.3042	0.00050

Palpitations	2	17	0.9135	1054	0.4882	0.00263

Swelling	2	95	0.4725	1050	0.2020	0.00914

Diabetes mellitus	2	69	0.7713	1073	0.1709	< 0.00001

Gini-Simpson relative diversities of selected attributes in 110 narrative and in 1119 structured medical reports for selected attributes categorized according to MDMC

**Table 3:** Gini-Simpson self diversities and relative marginal diversities

Attribute	Category	No. of narrative reports	Gini-Simpson self-diversity	Gini-Simpson relative marginal diversity	No. of structured reports	Gini-Simpson self-diversity	Gini-Simpson relative marginal diversity
Smoking	smoker	15	0.7887	0.6665	203	0.8120	0.6105
	
	occasional smoker	0	1.0000	0.0000	19	0.9824	0.0691
	
	ex-smoker	19	0.7324	0.7840	128	0.8815	0.4179
	
	nonsmoker	37	0.4789	0.9982	730	0.3241	0.8762

Allergy	yes	27	0.7	0.84	378	0.6451	0.9158
	
	no	63	0.3	0.84	681	0.3606	0.9222
	
	do not know	0	1.0	0.00	6	0.9944	0.0224

Ischemic heart disease	yes	56	0.1642	0.5489	44	0.9579	0.1613
	
	no	11	0.8358	0.5489	992	0.0507	0.1926
	
	I do not know	0	1.0000	0.0000	9	0.9914	0.0342

Dyspnoea	yes	28	0.6456	0.9152	113	0.8921	0.3851
	
	no	51	0.3544	0.9152	934	0.1079	0.3851

Chest pain	yes	10	0.7368	0.7756	87	0.9171	0.3042
	
	no	28	0.2632	0.7756	962	0.0829	0.3042

Palpitations	yes	6	0.6471	0.9135	150	0.8577	0.4882
	
	no	11	0.3529	0.9135	904	0.1423	0.4882

Swelling	yes	13	0.8632	0.4725	56	0.9467	0.202
	
	no	82	0.1368	0.4725	994	0.0533	0.202

Diabetes mellitus	yes	51	0.2609	0.7713	48	0.9553	0.1709
	
	no	18	0.7391	0.7713	1025	0.0447	0.1709

Gini-Simpson self diversities and relative marginal diversities in 110 narrative and 1119 structured medical reports for selected attributes categorized according to MDMC

**Figure 1:**
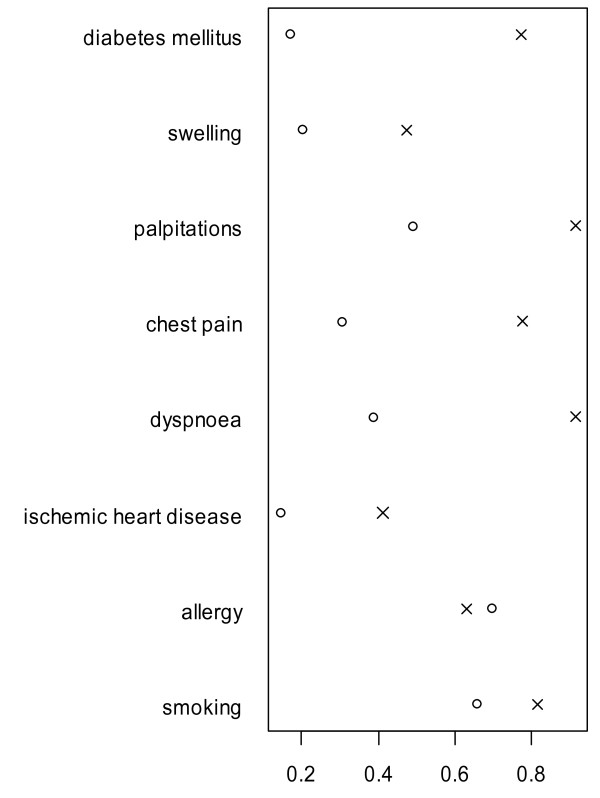
**Gini-Simpson relative diversities**. Gini-Simpson relative diversities in narrative (X) and structured (O) medical reports.

There were many missing values in attributes of narrative medical reports. We have suspected that these are non-recorded negative findings. We can see that except the Gini-Simpson relative diversity for the "Allergy" attribute, all calculated Gini-Simpson relative diversities in structured medical reports were significantly smaller at the 5% level (p < 0.05) than in narrative medical reports. The difference for the Allergy attribute is not significant at the 5% level. Statistical tests were performed using Z statistics with standardized normal distribution based on estimates of Gini-Simpson diversity. Figure 1 displays Gini-Simpson relative diversities of selected attributes categorized according to MDMC in narrative medical reports (X) and in structured medical reports (O). However, we assume that differences in diversities estimated from narrative and structured medical reports are caused also by many missing observations in narrative reports.

Finally, we compare percentage how often selected attributes appear in narrative and in structured medical reports and we achieve these results:

• *smoking *has been recorded in 64.5% of narrative and in 96.5% of structured medical reports;

• *allergy *in 81.8% of narrative and in 95.2% of structured medical reports;

• whether a patient suffers from an *ischemic heart disease *has been recorded in 60.9% of narrative and in 93.4% of structured medical reports;

• presence or absence of *dyspnoea *in 71.8% of narrative and in 93.6% of structured medical reports;

• whether a patient suffers from a *chest pain *has been found in 34.5% of narrative and in 93.7% of structured medical reports;

• questions about *palpitations *have been recorded only in 15.5% of narrative but in 94.2% of structured medical reports;

• *swelling *has been recorded in 86.4% of narrative and in 93.8% of structured medical reports and

• the *diabetes mellitus *attribute has been recorded in 62.7% of narrative and 95.9% of structured medical reports.

## Discussion

Close cooperation with physicians is essential for solving mapping problems. We consulted four physicians examining patients in both outpatient departments of preventive cardiology. It was often needed to choose the right synonym substituting a certain technical term. It was necessary to do it very carefully not to lose information or not to misinterpret it. In case that mapping is not possible without any lost of information, the better way is to describe a non-coded term by means of a set of several coded terms, possibly with showing mutual semantic relations. If this is not also possible, we can polemize with specialists whether these "indescribable" terms (attributes) can be replaced by other more equivalent or more standard ones. In special cases it is possible to add a certain term to an upcoming new version of a certain coding system. In case it is not possible to use any of the above mentioned possibilities of solving mapping problems, it is necessary to cope with the fact that mapping will never be 100%. The insufficient mapping process limits the interoperability of heterogeneous systems used for various purposes in healthcare. Restricted interoperability is often inevitable from the very root of the problem, e.g. insufficient harmonization of clinical contents of heterogeneous systems of electronic health records.

We can also see that while recording results of examinations by means of narrative medical reports terms for categories of attributes are not standardized and the Number of categories diversity is higher than for the same attribute in structured medical reports. Moreover, a lot of attributes in narrative medical reports are left unrecorded. It may have several reasons. Physicians do not have a strictly given skeleton according which they should proceed, so they may forget to collect some attributes. Another reason may be that physicians from the previous attributes know that the next attribute cannot be present and therefore they do not ask about it and they do not record it. But from the narrative medical reports we do not know whether these missing attributes have been checked or whether physicians on the basis of previous knowledge have deduced them. In structured medical reports (e.g. based on structured EHR) all attributes should be recorded. The application should not let physicians to continue if findings are not recorded. However, in our application based on MDMC, this was not the case and we can see that our physicians sometimes have not recorded some findings that could not be derived from other data.

## Conclusions

The new method for measuring diversity of medical reports can be applied to medical reports written in any language with categorized attributes. Moreover, it can quantitatively express possibilities for extraction of information from medical reports, more generally from any free text document. The analysis of narrative medical reports has shown that recording of attributes is very inaccurate and not standardized. The biggest problems for computational processing are typing errors, various length of shorten expressions and usage of synonyms. Another problem in the Czech healthcare is the lack of international classification systems translated to the Czech language. But even despite these problems in the usage of international classification systems in Czech healthcare, their use is a necessary first step to enable interoperability of heterogeneous systems of health records. Sufficient semantic interoperability of these systems is the basis for shared care, which leads to efficiency in healthcare, financial savings and reduction of the burden on patients. In this work we have tried to analyze how the international classification systems could be used best for the needs of Czech healthcare.

High diversity in narrative medical report leads to more difficult computer processing than in structured medical reports and some information may be lost during this process. Therefore it is very important to set standardized terminology that would be used in medical reports. Using international classification systems and nomenclatures we can compare diversities of medical reports written in the same or different languages among physicians or healthcare organizations.

The standardized terminology would bring many benefits to physicians. The standardized terminology would help to support development of electronic health records that can easily collect structured medical information. Structured medical reports have a smaller diversity and fewer numbers of missing observations. They can provide physicians, patients, administrators, software developers and payers with much more accurate and objective information. The standardized clinical terminology would help healthcare providers in a way that it could provide complete and easily accessible information that belongs to the process of healthcare (patient's medical record, diseases, treatments, laboratory results, etc.) and it would result in better care of patients.

## Competing interests

The authors declare that they have no competing interests.

## Authors' contributions

This paper is the result of numerous discussions among all three authors. Petra Přečková (corresponding author) contributed to the information lexical analysis of medical reports, interpretation of results, mapping Czech terminology to SNOMED CT and other classification systems, drafting the article and revising manuscript. Jana Zvárová contributed in giving advice and guide to the research design and data analysis, interpretation of results, revising and editing manuscript. Karel Zvára contributed to statistical evaluation of data, interpretation of results and revision of the manuscript. All authors read and approved the final draft.

## Pre-publication history

The pre-publication history for this paper can be accessed here:

http://www.biomedcentral.com/1472-6947/12/31/prepub
